# The Role of Tumor Microenvironment in Invasion and Metastasis of Esophageal Squamous Cell Carcinoma

**DOI:** 10.3389/fonc.2022.911285

**Published:** 2022-06-22

**Authors:** Shuyue Zheng, Beilei Liu, Xinyuan Guan

**Affiliations:** ^1^ Department of Clinical Oncology, Li Ka Shing Faculty of Medicine, The University of Hong Kong, Hong Kong, Hong Kong SAR, China; ^2^ Department of Clinical Oncology, The University of Hong Kong-Shenzhen Hospital, Shenzhen, China; ^3^ State Key Laboratory of Oncology in Southern China, Sun Yat-sen University Cancer Center, Guangzhou, China

**Keywords:** esophageal squamous cell carcinoma, tumor microenvironment, invasion, metastasis, immune regulation

## Abstract

Esophageal squamous cell carcinoma (ESCC) is one of the most common cancers in the world, with a high rate of morbidity. The invasion and metastasis of ESCC is the main reason for high mortality. More and more evidence suggests that metastasized cancer cells require cellular elements that contribute to ESCC tumor microenvironment (TME) formation. TME contains many immune cells and stromal components, which are critical to epithelial–mesenchymal transition, immune escape, angiogenesis/lymphangiogenesis, metastasis niche formation, and invasion/metastasis. In this review, we will focus on the mechanism of different microenvironment cellular elements in ESCC invasion and metastasis and discuss recent therapeutic attempts to restore the tumor-suppressing function of cells within the TME. It will represent the whole picture of TME in the metastasis and invasion process of ESCC.

## 1 Introduction

Esophageal squamous cell carcinoma (ESCC) is one of the most common cancers in the world, with high rates of morbidity and mortality (1). More than half of the ESCC patients are in advanced stages when they are first diagnosed. Extensive metastases prevent patients from having radical surgery, which is the only clinical method of curing ESCC currently (2). The Food and Drug Administration (FDA) has approved a number of new immune and targeted drugs, such as programmed cell death protein 1 (PD-1) inhibitors and human epidermal growth factor receptor-2 (Her-2) inhibitors for advanced ESCC treatment, but the survival rate of those advanced patients is still low (3, 4). It is reported that the 5-year survival rate for advanced esophageal cancer (19%) was on par with lung cancer (19%) and next only to liver cancer (18%) and pancreatic cancer (9%) (5, 6). Local invasion and distant metastasis of ESCC are the main reasons for the failure of treating these advanced patients. Therefore, further molecular research of the ESCC landscape has the potential to ascertain new biomarkers and molecular targets that affect ESCC progression and enable the design of new therapeutic strategies (7).

Recently, the central role of the tumor microenvironment (TME) in the invasion and metastasis of *de novo* ESCC has been identified. TME includes immune cells, fibroblasts, endothelial cells, perivascular cells, neurons, and extracellular matrix. There is increasing evidence that TME plays an important role in cell proliferation, cell survival, epithelial–mesenchymal transition (EMT), angiogenesis/lymphangiogenesis immunosuppression, invasion, and metastasis (8, 9). TME is a dynamic environment constantly reshaped by tumor and tumor-associated cells to make tumor cells survive well (10). Thus, TME is now regarded as a target-rich environment for the development of novel anticancer drugs in ESCC. Actually, many drugs that focus on diverse components of TME, including vascular endothelial growth factor (VEGF) and immune checkpoints, have been approved for clinical use (11, 12).

In this review, we summarize recent advances in how ESCC cells recruit and modify cells in the immune microenvironment to make them more conducive to metastasize and how those factors in the TME support the ESCC invasion and metastasis. Also, we discuss the regulation of abnormal molecular signaling pathways and networks stimulated by tumor and TME interactions, which might provide new diagnostic, prognostic, or therapeutic opportunities.

## 2 Invasion and Metastasis Process of Esophageal Squamous Cell Carcinoma

**Figure 1:**
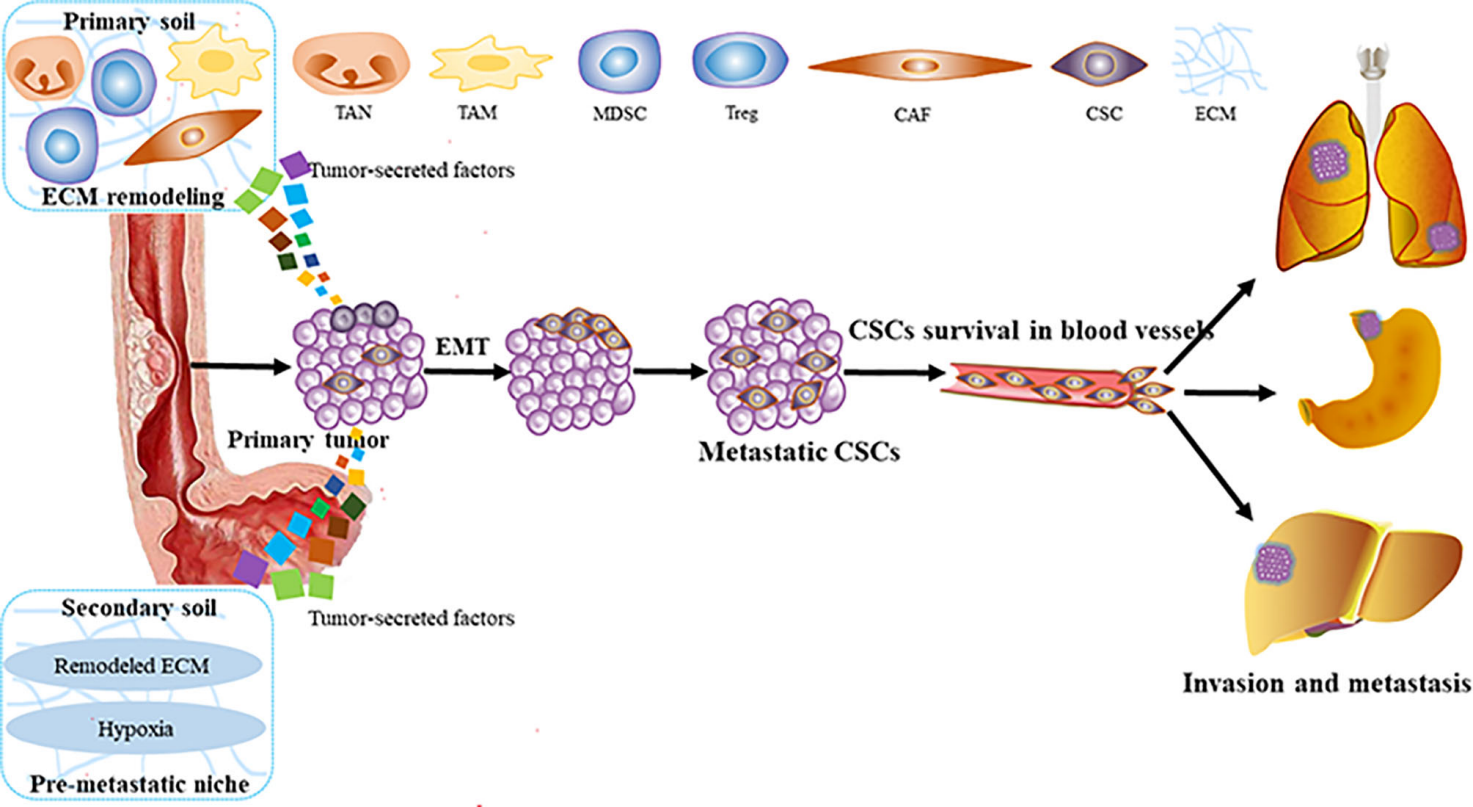
The process of ESCC invasion and metastasis. ESCC, esophageal squamous carcinoma; MDSC, marrow-derived suppressor cell; Tregs, regulatory T cells; CAFS, cancer associated fibroblasts; EMT, epithelial—mesenchymal transition; TAM, tumor-associated macrophage; TAN, tumor-related neutrophil; ECM, extracellular matrix; CSCs, cancer stem cells.

Metastasis is the process by which circulating tumor cells colonize in other tissues or organs and become diffuse tumor cells. However, only 0.01% of circulating tumor cells have been reported to successfully colonize and grow into diffuse tumor cells (13, 14). It is because the circulating tumor cells are seriously influenced by the human local microenvironment. The “seed and soil” hypothesis raised by Paget can be used to well characterize this process (15). Tumor cells *in situ* (“seeds”) tend to stay on some specific target organs (“soil”), which have TME beneficial to the survival of tumor cells. At present, it is supposed that there are three main steps for the formation of a metastasis niche: first, the primary tumors secrete some factors around them (invasion), exosomes, and micro-vesicles (metastasis) to create the pre-metastatic niche (16, 17). Then, those factors induce immune cells, such as marrow-derived suppressor cells (MDSCs), macrophages, dendritic cells (DCs), neutrophils (18, 19), and regulatory T cells (Tregs) to polarize into tumor-promoting cells. Also, some stromal components such as cancer-associated fibroblasts (CAFs) promoting angiogenesis, secreting cytokines, inducing EMT, recombining matrix components, recruiting inflammatory cells to help ESCC cells invade and metastasize (20, 21), and other factors (hypoxia, etc.) (17). Finally, all those factors remodel the microenvironment into TME, and invasion and metastasis occur (**Figure 1**).

Lymphatic metastasis is the most common way of ESCC metastasis, which is determined by the characteristics of lymphatic reflux in the esophageal wall (22, 23). Also, lymph node metastasis is the most important prognostic factor of ESCC. As to the “seeds and soil” hypothesis, lymph node metastasis is not a simple process of direct migration of ESCC cells. Many kinds of literature have reported that the niche of ESCC lymph nodes has changed significantly before metastasis (24, 25). It has been shown that the lymph node immune status of pN0 and pN1 patients is completely different. There is an obvious activated pattern of immune response in the pN0 patients. On the contrary, pN1 patients show a distinct pattern of inhibition, such as reduced immune response, immune cell proliferation, and increased immune cell apoptosis (26, 27). It means that in the early stage of ESCC metastasis, drainage of tumor antigens to lymph nodes results in the antitumor status. However, as time goes on, more and more tumor secretory factors and immunosuppressive cells will accumulate. Then, the immune state of lymph nodes will change from antitumor to pro-tumor mode until the tumor cells first colonize and metastasize (28). Therefore, an in-depth study of the interaction between tumor cells and the immune microenvironment and how it promotes the ESCC invasion and metastasis will guide the development of future diagnosis and treatment strategies.

## 3 The Role of Tumor Microenvironment in Esophageal Squamous Cell Carcinoma Invasion and Metastasis

### 3.1 Immune Modulation Promotes Esophageal Squamous Cell Carcinoma Invasion and Metastasis

**Figure 2:**
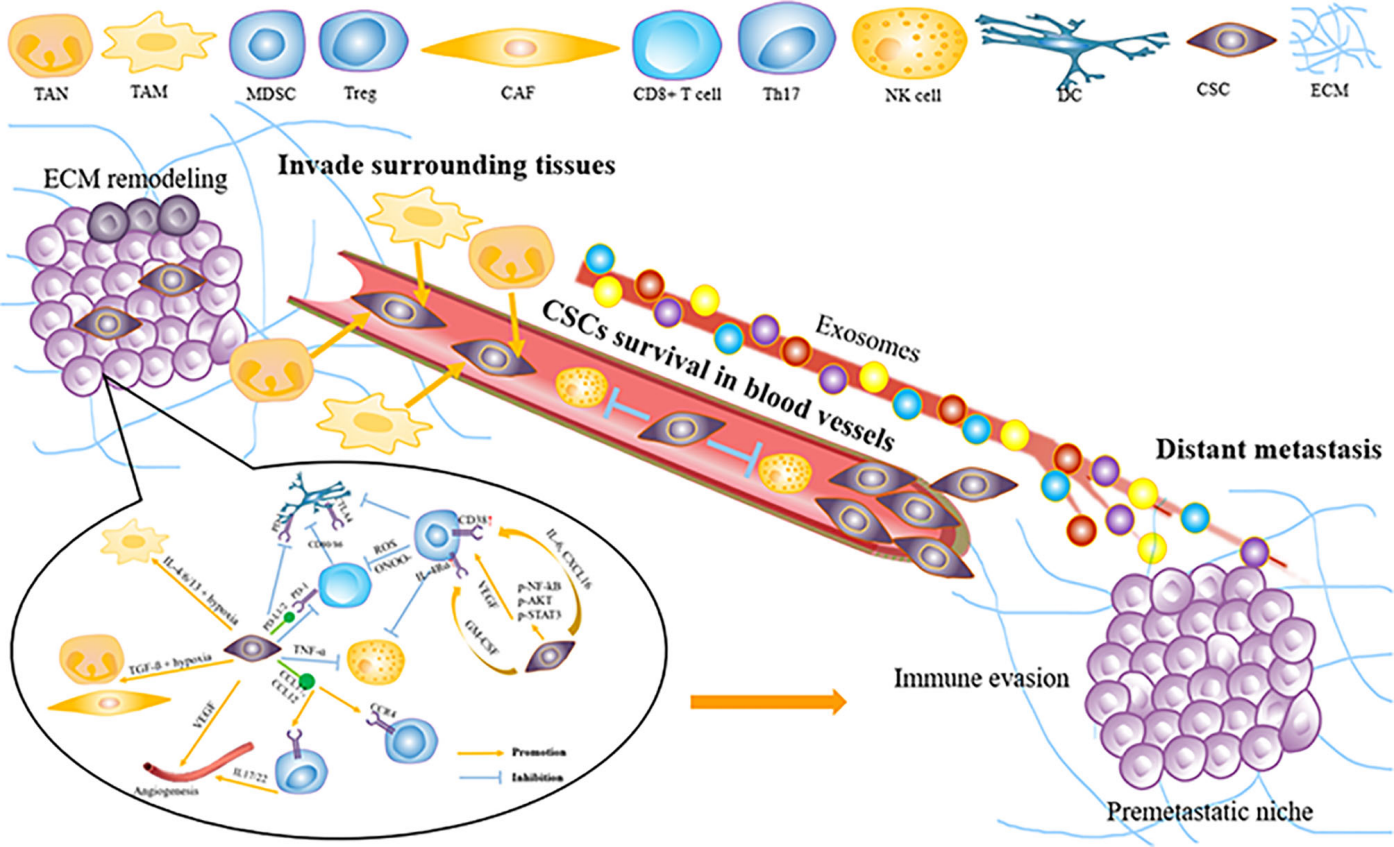
How tumor microenvironment support ESCC invasion and metastasis. ESCC, esophageal squamous carcinoma; MD.SC, man-our-derived suppressor cell; DC, dendritic cell; Treg, regulatory T cell; CAE, Cancer associated fibroblast; VEGF, vascular endothelial growth factor; NK cell, natural killer cell; TAM, tumor-associated macrophage; TAN, tumor-related neutrophil; ECM, extracellular matrix; CSCs, cancer Stem cells.

Tumors escaping from the immune system are the key to tumor invasion and metastasis. Tumor cells can form specific TME that inhibits antitumor immune response by recruiting various alternative tumor-associated immune cells or expressing inhibitory molecular factors (**Figure 2**). Specific immune cell types and influencing factors in ESCC will be discussed below.

#### 3.1.1 Myeloid-Derived Suppressor Cells

Myeloid-derived suppressor cells (MDSCs) are the suppressive cell population of the immune system, which play a pivotal role in the TME (29). MDSCs can greatly inhibit the cytotoxic function of T cells and NK cells during circulation and support ESCC progression (30). The specific markers of MDSCs are most often identified by the expression of CD11b and lack of HLA-DR expression (31). In ESCC, MDSCs produce reactive oxygen species (ROS) and peroxynitrite (ONOO^−^), which block the activation and proliferation of T cells to disrupt immune responses (32). Also, MDSCs inhibit the proliferation of CD8^+^ T cells by phosphorylating T-cell receptor (TCR) and CD8 molecules during direct interaction with T cells, which results in the downregulation of immune activity (33, 34). In addition, VEGF produced by MDSCs promotes tumor angiogenesis, creates a pre-metastasis environment, and prolongs immunosuppression (35, 36). Furthermore, it has been demonstrated recently that MDSCs could paralyze T cells by cell–cell transfer of the metabolite methylglyoxal, which would reduce the antitumor immunity of T cells and promote invasion and metastasis (37). Further research into the biology of MDSCs, especially the functions of specific population cells, will provide directions for therapeutic development.

#### 3.1.2 Regulatory T Cells

Tregs, a subgroup of CD4^+^ helper T cells identified by CD25 and Foxp3 expression, play an immunosuppressive role in cancer. Tregs attenuate antitumor immunity by secreting immunosuppressive cytokines, interfering with tumor-associated antigen presentation, and inhibiting cytotoxic cell function (38, 39). It has been demonstrated that Foxp3 expression in ESCC means a poor prognosis (40, 41). It is reported that FOXP3 might directly inhibit the IL-2 and promote cytotoxic T lymphocyte-associated antigen 4 (CTLA4) and CD25 expression (42). In ESCC, increased recruitment of Tregs is mediated, at least in part, by chemokines CCL17 and CCL22, secreted by tumor cells and macrophages (43). It has been reported that IL-33, which has a high expression in ESCC, could promote CCL2 expression *via* the NF-κB pathway and then recruit Tregs to promote ESCC migration (44, 45). Treg infiltration has been found to be prognostic, and more Tregs are often associated with deeper tumor invasion, extensive metastasis, and reduced survival (46, 47). Tregs have several context-dependent functions that are not well described, which poses challenges for ESCC invasion and migration.

#### 3.1.3 Tumor-Associated Macrophages

Tumor-associated macrophages (TAMs) promote various pro-tumor mechanisms. Macrophages are classified into M1 and M2 types, of which M2 macrophages secreted type II cytokines to facilitate various pro-tumorigenic mechanisms (48). The specific markers of TAMs are most often identified by expression of iNOS for M1 type and CD163 for M2 type. Hypoxia can induce M2 polarization, and then TAMs will produce growth factors and proteases that promote tumorigenesis and inhibit the immune system, angiogenesis, invasion, and metastasis (49, 50). CD68^+^ PD-1^+^ TAMs in ESCC TME tend to be of M2 phenotype, which can result in the upregulation of PD-L1 expression in tumor cells and promote ESCC invasion and migration (51, 52). Activation of the AKT/ERK pathway is a driving force for ESCC cell invasion and migration, and this pathway can be triggered by a variety of factors produced by TAMs or cancer cells themselves (53, 54). CD163^+^ TAMs can also promote ESCC cell invasion and migration by releasing thymidine phosphorylase (TP) to augment angiogenesis and produce IL-1β to enhance EMT (55, 56). The M2/M1 macrophage ratio of ESCC patients has also been used as a predictor of lymph node metastasis (57). All of these suggest potential intervention and immunotherapy strategies for TAMs in the invasion and migration of ESCC patients.

#### 3.1.4 Tumor-Associated Neutrophils

Tumor-associated neutrophils (TANs) are completely different from circulating neutrophils (58). Transforming growth factor-β (TGF-β) in TME promotes the transformation of neutrophils from antitumor N1 to pro-tumor N2 (59). Unlike M1 and M2, there is no suitable marker to indicate the N1 and N2 neutrophils in the tumor (60). The study of TANs mainly focuses on the neutrophil-to-lymphocyte ratio (NLR) (60). It has been reported that preoperative NLR elevation was associated with lymph node metastasis, deeper tumor invasion, and advanced TNM stage (61). Neutrophils will undergo apoptosis after activation, forming neutrophil extracellular traps (NETs), which have been shown to predict the lymph node and distant metastasis (62, 63). All of these indicate that TANs can be a good predictor of ESCC invasion and migration.

#### 3.1.5 Mast Cells and Eosinophils

Mast cells (MCs) and eosinophils often co-participate in response to parasitic infections and allergic diseases (64). In the TME of ESCC, high MC density has been found to be closely associated with tumor angiogenesis, invasion, and metastasis and predicts poor survival in ESCC patients (65, 66). It is reported that trypsin release from MCs promotes tumor cell metastasis through exosomes (67). Yet the high expression of eosinophils has been reported to be positively associated with low rates of metastasis in early ESCC patients (68). Also, it has been reported recently that metastasis-entrained eosinophils could promote lymphocyte-mediated antitumor immunity (69). A large number of new studies are needed for the mechanism of eosinophil in ESCC, which will provide new ideas for the ESCC invasion and metastasis and eosinophil-based immunotherapy.

#### 3.1.6 Th17 Lymphocytes

Th17 lymphocytes are a branch of CD4^+^ helper T cells, and IL-17 is its main effector molecule. IL-17A expressed by Th17 cells can induce the production of chemokines in ESCC cells, such as CCL20, CXCL-9, CXCL-10, and CXCL13 (70, 71). These chemokines could promote the proliferation and differentiation of Th17 lymphocytes in ESCC TME (72). Also, increased Th17 lymphocytes are positively associated with more lymph node metastasis (73). It has been reported that IL-17A can activate MMP-2 and MMP-9 through the ROS/NF-κB signaling pathway (74), while matrix metalloproteinases (MMPs) could catalyze the degradation of extracellular matrix and promote ESCC migration and metastasis (75, 76). The role of Th17 lymphocytes in ESCC invasion and metastasis needs to be further investigated.

### 3.2 Stromal Components Facilitate Esophageal Squamous Cell Carcinoma Invasion and Metastasis

In addition to immune cells, stromal components and CAFs play a critical role in ESCC invasion and metastasis (77) (**Figure 2**). Fibroblast activation protein-α (FAP) and α-smooth muscle actin (α-SMA) are often used as the markers for the activated phenotype of CAFs, of which the process is induced by ESCC secreting TGF-β (78, 79).

CAFs have been proved to promote ESCC invasion and metastasis by secretion of cytokines, induction of EMT, recruitment of immune cells, and other mechanisms to reconstruct TME (80). IL-6 secreted by FAP^+^ CAFs not only can promote ESCC cell invasion and EMT but also can recruit FoxP3^+^ T cells and induce TAM M2 polarization to promote metastasis (81, 82). The presence of CAFs in ESCC patients is associated with increased micro-vessel density, TAMs, and EMT, which are critical for ESCC invasion and metastasis (83, 84). A number of genes have been shown to promote ESCC invasion and metastasis *via* the CAF transformation and EMT process (85, 86). Also, it has been demonstrated that CAFs promote ESCC invasion by secreting hepatocyte growth factor (HGF) and infiltrating MDSCs (87, 88). Also, CAFs have been reported to be associated with low 3-year survival and ESCC progression after chemoradiotherapy (89). FAP-α has been reported to be an important regulator in ESCC lymph node metastasis (90). HGF and TGF-β are closely related to tumor invasion and metastasis (91). It has been demonstrated that CAFs could express HGF and TGF-β1 and then promote ESCC invasion and metastasis *via* the HGF/Met and TGFβ1/Smad pathways, respectively (92, 93). It has been confirmed that infiltrating MDSCs activate CAFs to promote ESCC invasion (94). Interaction between CAFs and immune cells to promote ESCC invasion and metastasis needs further research.

Due to the high heterogeneity of ESCC, traditional genomic and transcriptome analyses tend to ignore some signals displayed by specific cell populations or cell states. However, with the development of single-cell sequencing technology, several single-cell studies about ESCC and TME have been published in recent years. It has been reported that single-cell transcriptome sequencing was performed in 11 ESCC patients to analyze the TME. Heterogeneity was found in most ESCC interstitial cell types, particularly between fibroblasts and immune cells. Also, tumor-specific CST1^+^ myofibroblast subpopulations had been identified to have prognostic values and potential biological significance (95). Also, the main association framework between cancer cells and various non-cancer cells in TME has been established *via* single-cell transcriptome sequencing, which contributes to the further investigation of ESCC progression and prognosis (96). Furthermore, a comparison between esophagus non-malignant tissues and ESCC tissues *via* single-cell transcriptome network analysis has shown that energy supply-related pathways are pivotal in cancer metabolic reprogramming for TME. Immune checkpoints, which are potential targets for ESCC immunotherapy, have been found to be significantly overexpressed in ESCC, including LAG3 and HAVCR2 (97). At present, there are no single-cell studies specifically for ESCC invasion and metastasis, which needs further investigation.

## 4 The Role of Cellular Communication in Esophageal Squamous Cell Carcinoma Invasion and Metastasis

### 4.1 Tumor Cells Remodel Tumor Microenvironment to Promote Esophageal Squamous Cell Carcinoma Invasion and Metastasis

#### 4.1.1 Cytokine/Chemokine Network

Metastasis is a multistep process that requires tumor cells to separate from the primary tumor and migrate through the lymphatic or blood circulatory system to target distant organs (98). There is increasing evidence that primary tumors can prepare the cytokine/chemokine network for invasion and metastasis (99, 100) (**Figure 2**).

CXCL12 is a chemokine that functions through CXCR4 and plays an important role in ESCC invasion and metastasis (101). It is noteworthy that CXCR4 is expressed only in ESCC tissues but not in the normal esophageal epithelium (102). Expression of CXCL12 or CXCR4 in ESCC patients is significantly related to ESCC invasion, lymph node metastasis, and poor survival (103, 104). It has been shown that ESCC cells could secrete large amounts of CXCL12 *via* an autocrine way and increase their receptor CXCR4 expression compared with normal cells (105). Also, ESCC cells could enhance the activation of the p-ERK1/2 pathway *via* the CXCL12/CXCR4 axis to promote ESCC invasion and metastasis (106).

It has been reported that CCR7, combined with CCL21, supports a metastatic niche directly (107). A number of studies have shown that high levels of CCR7 are related to ESCC metastasis and poor survival (108). It has been investigated that co-expressed CCR7 and MUC1 could facilitate ESCC invasion and metastasis *via* the ERK1/2 pathway (109, 110). Some studies have also demonstrated that there is an interaction between CCR7 and VEGF-C, and their expression can be used as the predictor for ESCC lymphatic metastasis (111).

Many studies have indicated that high levels of CXCL8 and CXCR2 in ESCC patients are associated with metastasis and poor prognosis (112). It has been shown that CXCL8 is upregulated in TAMs and promotes ESCC invasion and metastasis *via* CXCR1/CXCR2 receptors to activate AKT and ERK1/2 signaling pathways (52). Also, a clinical study has shown that CXCL8 expression is significantly associated with metastasis and the increase of CXCR2- and CD204-positive macrophages (108, 113). It is necessary to further investigate the biological significance of cytokine/chemokine networks in ESCC and their potential use as future drug targets.

#### 4.1.2 Exosome

Exosomes are nanovesicles (30–150-nm diameter) that are secreted by various cell types (114). Recently, it has been shown that exosomes play important roles in ESCC invasion and metastasis (115). It is reported that exosomes released by ESCC can enrich miR-320b and promote ESCC lymph node metastasis *via* programmed cell death 4 (PDCD4) through the AKT signaling pathway (116). Exosome-shuttling miR-21 has been shown to promote ESCC invasion and metastasis by targeting PDCD4 *via* the c-Jun N-terminal kinase (JNK) signaling pathway (117). Clinical data have also displayed that serum exosomal hsa_circ_0026611 expression is significantly upregulated with ESCC lymph node metastasis (118). Exosome long non-coding RNA (lncRNA) LINC01711 promotes ESCC invasion *via* FSCN1 upregulation and miR-326 downregulation (119). Also, it has been reported that T cell-derived exosomes promote ESCC metastasis *via* promoting EMT by β-catenin and NF-κB/snail signaling pathways upregulation (120). However, there is still a long way to the mechanisms of how these exosomes are involved in ESCC invasion and metastasis.

#### 4.1.3 Vascular Endothelial Growth Factor

VEGF is the key mediator of angiogenesis, which has the function of triggering endothelial cell proliferation, migration, and breakdown of the extracellular matrix for new blood vessels. It has been reported that when tumor cells overexpressing HMGB1 co-cultured with B cells, the proliferating B cells can be induced to express VEGF and then elevate angiogenesis (121). A significant decrease in VEGF-C has been found in high tumor lymphocytic infiltration (122). It is reported that low expression of CD80 can be associated with VEGF overexpression. CD80 impairment in the ESCC tissues is correlated with poor survival, which indicates the dysfunction of the immune system and promotes the ESCC progression (123). Some studies have confirmed that VEGF-C, a lymphangiogenic factor, is associated with survival, tumor depth, stage, and lymph node metastasis of ESCC (124, 125). Also, many genes have been reported to promote ESCC invasion and metastasis *via* VEGF-related pathways or axis (126, 127). Development of new angiogenesis inhibitors and regulation of tumor vascular microenvironment are still possible ways to treat ESCC invasion and metastasis.

### 4.2 The Interaction Between Immune Cells Promotes Esophageal Squamous Cell Carcinoma Invasion and Metastasis

In addition to the interaction between various immune cells and ESCC cells, there is an important interaction among various immune cells, which indirectly promotes ESCC invasion and metastasis. For example, Th-2 could secrete many cytokines (IL-6 and IL-13) to recruit MDSCs in the ESCC TME (128, 129). Also, IL-4 and IL-13 derived from Th-2 could promote macrophages polarizing into M2 macrophages (130). MDSCs with high CD38 levels have been reported to inhibit the cytotoxic effect of ESCC-activated T cells (131). MDSCs could also induce Tregs and CAFs to inhibit the antigen-presenting cells (APCs) and indirectly inhibit the cytotoxic effect of ESCC-activated T cells (42, 132). In addition, ESCC cells could produce RCAS1 to induce DC, promote tumor-infiltrating lymphocyte apoptosis, and inhibit CD8^+^ T-cell activity (133). IL-17A-producing cells could enhance CD1a^+^ DC infiltration of TME *via* the release of CCL2 or CCL20, which is associated with better survival in ESCC patients (134). Th17 cells and MCs in ESCC TME have been shown to secrete IL-17 to promote ESCC cells to release CXCL9/10, CXCL2/3, and CCL2/20, which could facilitate NK cell infiltration and activity (66). PD-1, a member of the CD28 family, is mainly expressed on activated T cells (135). When PD-1 is combined with its ligand (PD-L1 or PD-L2), which can be expressed by tumor cells, immune cells (i.e., macrophages), and endothelial cells, then T-cell activation will be inhibited (136, 137). TME contains a variety of immune cells, which form a complex regulatory network through receptor-ligand binding or the release of various immune factors, thus affecting the invasion and metastasis of ESCC.

## 5 Targeting Tumor Microenvironment for Esophageal Squamous Cell Carcinoma Invasion and Metastasis

**Figure 3:**
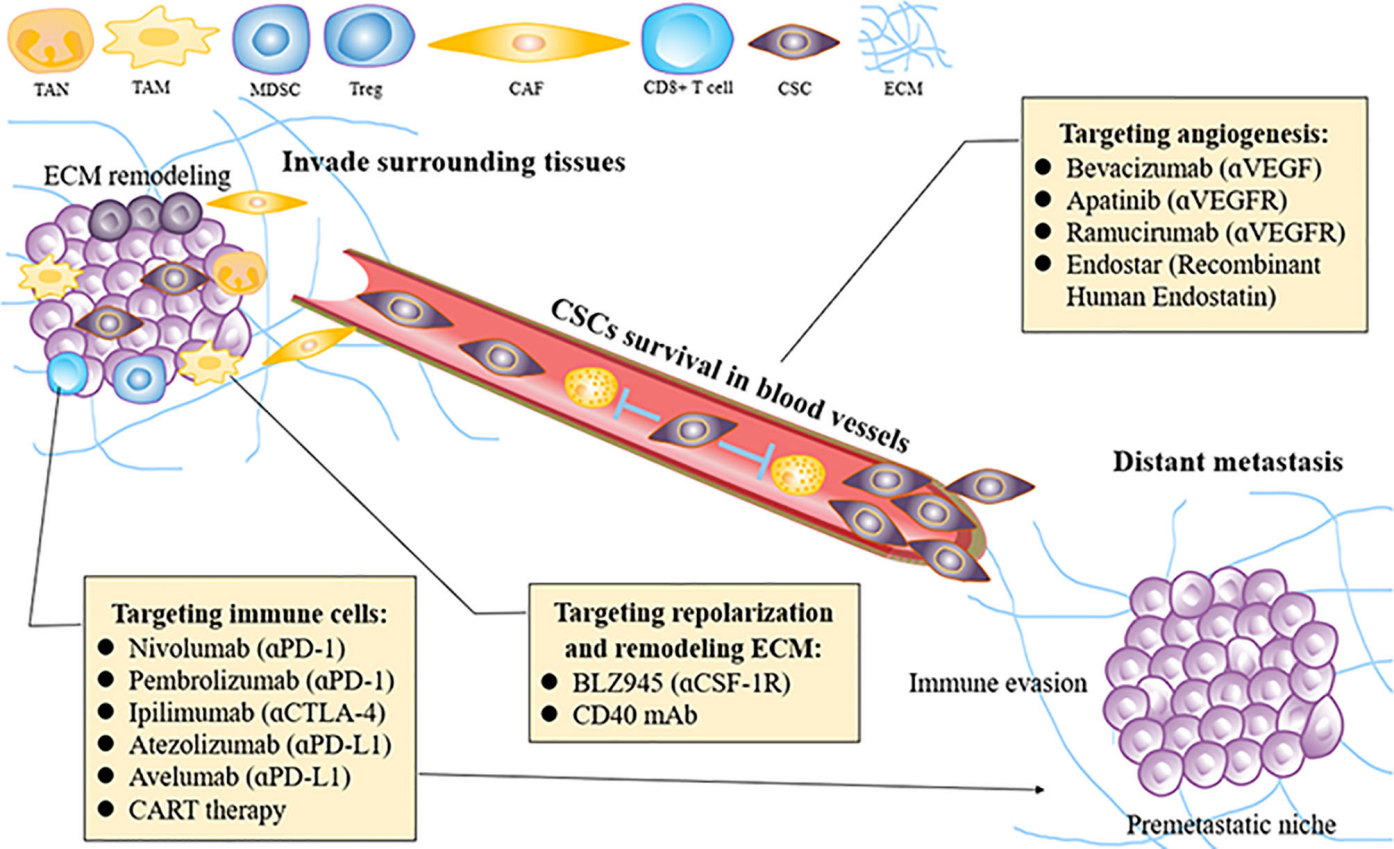
How to re-educate the tumor microenvironment for treating ESCC invasion and metastasis. ESCC, esophageal squamous carcinoma; MDSC, mallow-derived suppressor cell; Treg, regulatory T cell; CAF, cancer associated fibroblast; VEGF, vascular endothelial growth factor; TAM, tumor-associated macrophage; TAN, tumor-related neutrophil; ECM, extracellular matrix; CSCs, cancer stem cells; PID-1, programmed cell death protein 1; CTLA4, T lymphocyte-associated antigen 4; CSF-1, colony-stimulating factor-1.

Targeting approaches using different methods to remodel the TME and then inhibit ESCC invasion and metastasis are discussed as follows (**Figure 3**).

### 5.1 Targeting Angiogenesis for Esophageal Squamous Cell Carcinoma Invasion and Metastasis

Angiogenesis plays a crucial role in the development of ESCC, by delivering oxygen and nutrients to tumors, and its key mediator is VEGF (138). Distant vascular metastasis is another way of tumor progression. Many VEGF/VEGFR inhibitors have been developed to induce vascular normalization and make patients more sensitive to chemotherapy (139). It has been found that low doses of VEGF inhibitor (apatinib) could regulate the TME, relieve hypoxia, and increase the number of T cells at the tumor site, thereby enhancing the efficacy of PD-1/PD-L1 inhibitors, while excessive doses do not produce such an effect (140). However, this theory has not been tested in ESCC. The development of new angiogenesis inhibitors and regulation of vascular TME are still possible ways to avoid ESCC invasion and metastasis.

### 5.2 Targeting Immune Markers for Esophageal Squamous Cell Carcinoma Invasion and Metastasis

#### 5.2.1 Immune Checkpoint Inhibitors

PD-1 is an immune checkpoint that inactivates T-cell immune function. Its two ligands, PD-L1 and PD-L2, combined with the PD-1 receptor, could induce depletion of PD-1 signaling pathways and associated T cells and inhibit T-cell activation and proliferation reversibly (141). Many studies have reported that the expression of both PD-L1 and PD-L2 is elevated in ESCC. In fact, in ESCC patients, increased PD-L1 or PD-L2 expression in ESCC cells is correlated with reduced survival, while increased PD-L1 expression is associated with increased depth of tumor invasion and worse survival (142, 143). In addition, the expression of PD-L2 is related to decreased CD8^+^ T-cell infiltration. The increased PD-L2 expression is induced by tumor-promoting Th2 cytokines such as IL-13 or IL-4 (144).

The expression of CTLA4 is another immune checkpoint that inactivates by inhibiting TCR signaling (145). CTLA4 is expressed not only in tumor-infiltrating immune cells but also in cancer cells, which is a key part of immune escape (146). Existing evidence already suggests that PD-1 inhibitors show therapeutic promise in lung cancer and melanoma and might also be used in ESCC (147). Also, many studies are targeted at how to regulate other immune cells in TME to improve the efficacy of immunotherapy (148, 149).

#### 5.2.2 Other Immune Cells

TAMs can produce a variety of tumor-promoting factors, such as colony-stimulating factor-1 (CSF-1), so they might be attractive targets for remodeling immune responses within TME (150). In recent years, targeting TAM therapies such as CSF-1 or CSF-1R blockade have attracted extensive attention in tumor research. The combination of CSF-1R blockade and PD-1/PD-L1 inhibitors is underway (NCT02323191) (151). IL-6 secreted by FAP^+^ CAFs not only can promote ESCC cell invasion and EMT but also can recruit FoxP3^+^ T cells and induce TAM M2 polarization to promote metastasis (81, 82). Using CAF-targeted NIR-PIT to eliminate CAFs could interfere with ESCC invasion and metastasis effectively. The combination of the CAF-targeted NIR-PIT with traditional anticancer drugs might be a promising choice (152).

### 5.3 T-Cell Modification for Esophageal Squamous Cell Carcinoma Invasion and Metastasis

Chimeric antigen receptor (CAR) T-cell therapy means that T cells are modified into CAR T cells by genetic engineering to specifically recognize and attack tumor cells (153). Ephrin type A receptor 2 (EphA2) and HER-2, highly expressed in ESCC, are common targets of CAR T-cell therapy and have been verified to effectively kill esophageal cancer cells (154, 155). Enhanced MUC1-CAR T cells have been shown to have better antitumor activity because they can survive longer *in vivo*, which means they have long-lasting antitumor effects (156). Also, it has been recently reported that IDO1 inhibitor-loaded nanosheets could enhance CAR T-cell effectiveness in ESCC and CD276 suppress CAR T-cell function (157, 158). The selection of different solid tumor-specific antigens and the delivery of CAR T cells are still the disadvantages of CAR T-cell therapy (159).

## 6 Conclusion

In this review, we have summarized how ESCC invasion and metastasis occur and discussed how the major cell populations, stromal components, and their interaction in the TME promote ESCC invasion and metastasis. Also, we summarized recent therapies targeting TME for ESCC invasion and metastasis. Looking forward, it is critical to further investigate how cancer cells transfer to the new environment and adapt surrounding cells and components into a suitable environment for tumor invasion and metastasis. At present, there are few diagnostic methods and new drugs targeted for ESCC invasion and metastasis. Advances in these areas promise improved treatment options and better outcomes for this deadly disease.

## Author Contributions

XYG and BLL supervised and reviewed the manuscript. SYZ conducted the literature review and wrote the manuscript. All authors contributed to the article and approved the submitted version.

## Funding

This work was supported by grants from the Hong Kong Research Grant Council (RGC) grants including Collaborative Research Funds (C7065-18GF, C7026-18GF, and C4039-19GF), Research Impact Funds (R4017-18, R1020-18F, and R7022-20), Shenzhen Fundamental Research Program (JCYJ20180508153249223), The Shenzhen Science and Technology program (KQTD20180411185028798), and the Program for Guangdong Introducing Innovative and Entrepreneurial Teams (2019BT02Y198). XYG is the Sophie YM Chan Professor in Cancer Research.

## Conflict of Interest

The authors declare that the research was conducted in the absence of any commercial or financial relationships that could be construed as a potential conflict of interest.

## Publisher’s Note

All claims expressed in this article are solely those of the authors and do not necessarily represent those of their affiliated organizations, or those of the publisher, the editors and the reviewers. Any product that may be evaluated in this article, or claim that may be made by its manufacturer, is not guaranteed or endorsed by the publisher.
